# Coleon U, Isolated from *Plectranthus mutabilis* Codd., Decreases P-Glycoprotein Activity Due to Mitochondrial Inhibition

**DOI:** 10.3390/pharmaceutics15071942

**Published:** 2023-07-12

**Authors:** Sofija Jovanović Stojanov, Epole N. Ntungwe, Jelena Dinić, Ana Podolski-Renić, Milica Pajović, Patrícia Rijo, Milica Pešić

**Affiliations:** 1 Institute for Biological Research “Siniša Stanković”—National Institute of the Republic of Serbia, University of Belgrade, 11060 Belgrade, Serbia; sofija.jovanovic@ibiss.bg.ac.rs (S.J.S.); jelena.dinic@ibiss.bg.ac.rs (J.D.); ana.podolski@ibiss.bg.ac.rs (A.P.-R.); milica.pajovic@ibiss.bg.ac.rs (M.P.); 2 CBIOS (Research Center for Biosciences and Health Technologies), Universidade Lusófona de Humanidades e Tecnologias, Campo Grande 376, 1749-024 Lisbon, Portugal; p5999@ulusofona.pt; 3 Department of Chemistry, University of Coimbra, P-3004-535 Coimbra, Portugal

**Keywords:** Coleon U, P-glycoprotein, cancer multidrug resistance

## Abstract

Multidrug resistance in cancer is often mediated by P-glycoprotein. Natural compounds have been suggested as a fourth generation of P-glycoprotein inhibitors. Coleon U, isolated from *Plectranthus mutabilis* Codd., was reported to modulate P-glycoprotein activity but the underlying mechanism has not yet been revealed. Therefore, the effects of Coleon U on cell viability, proliferation, and cell death induction were studied in a non-small-cell lung carcinoma model comprising sensitive and multidrug-resistant cells with P-glycoprotein overexpression. P-glycoprotein activity and mitochondrial membrane potential were assessed by flow cytometry upon Coleon U, sodium-orthovanadate (an ATPase inhibitor), and verapamil (an ATPase stimulator) treatments. SwissADME was used to identify the pharmacokinetic properties of Coleon U, while P-glycoprotein expression was studied by immunofluorescence. Our results showed that Coleon U is not a P-glycoprotein substrate and is equally efficient in sensitive and multidrug-resistant cancer cells. A decrease in P-glycoprotein activity observed with Coleon U and verapamil after 72 h is antagonized in combination with sodium-orthovanadate. Coleon U induced a pronounced effect on mitochondrial membrane depolarization and showed a tendency to decrease P-glycoprotein expression. In conclusion, Coleon U-delayed effect on the decrease in P-glycoprotein activity is due to P-glycoprotein’s functioning dependence on ATP production in mitochondria.

## 1. Introduction

Non-small-cell lung cancer (NSCLC) is a common and highly lethal tumor worldwide [1]. Most NSCLC patients are diagnosed at an advanced stage of the disease; however, despite extensive research, the intrinsic and acquired chemotherapy resistance, particularly multidrug resistance (MDR), remains a significant challenge in improving treatment outcomes, often leading to treatment failure and disease relapse [2]. Statistical data indicate that drug resistance is a major contributor to over 90% of cancer patient mortality [3].

During chemotherapy, MDR can occur through a variety of mechanisms, including enhanced drug efflux, genetic factors (such as gene mutations, amplifications, and epigenetic changes), growth factors, increased DNA repair capacity, and increased metabolism of xenobiotics [3,4,5,6,7]. Each of these mechanisms decrease the effectiveness of administered drugs, making tumor treatment more challenging. P-glycoprotein (P-gp), an ATP-binding cassette (ABC) transporter, plays a crucial role in drug resistance [8]. P-gp is located in the cell membrane and regulates the distribution, absorption, and excretion of various chemical compounds [9]. It is highly expressed in cancer cells and functions as an efflux pump, actively removing drugs from the cells, leading to decreased effectiveness and contributing to MDR [3]. Developing new inhibitors that can inhibit P-gp function or discovering antitumor drugs that are not P-gp substrates is essential to prevent drug efflux and increase the sensitivity of cancer cells to drugs.

Natural products have gained attention as agents with the potential to overcome MDR [10]. Various compounds of natural origin, including flavonoids, alkaloids, coumarins, and terpenoids, have been investigated as potential modulators of P-gp activity [11,12,13,14]. Terpenoids, specifically abietane-type diterpenoids found in the *Plectranthus* genus, have been identified as inhibitors of P-gp activity [15]. *Plectranthus mutabilis* Codd. is a succulent plant from South Africa that is threatened with extinction, and its extract contains compounds with cytotoxic activity against various cancer cell lines [15,16,17]. Examining this rare plant with significant medicinal potential can raise awareness of its protection and conservation. Our work in the last few years is partly dedicated to this goal. Coleon U, an abietane diterpenoid, is the prominent diterpenoid in *Plectranthus mutabilis* Codd. extract (Coleon U is the most abundant compound with 96 μg/mg in the acetone extract from the leaves of *Plectranthus mutabilis* Codd.) [17]. The available literature on the Coleon U mode of action in cancer is limited and this motivated us to study its anticancer mechanism of action. It was discovered that Coleon U can impact the immune system’s functional status by inhibiting the proliferation of human lymphocytes, making Coleon U a promising candidate for the development of immunomodulatory drugs [18]. Coleon U has also been shown to inhibit the growth of several human cancer cell lines: breast, central nervous system, kidney, and melanoma [19]. Although its precise mechanisms of action are not fully understood, Coleon U selectively activates a specific isoform of protein kinase C (PKC-δ) involved in apoptotic pathways, making it a promising candidate for further evaluation as an anticancer drug [20]. Additionally, Coleon U has shown the potential to reverse resistance to doxorubicin (DOX) in MDR NSCLC cells and increase its efficacy [17].

This study aims to elucidate the mechanism underlying the Coleon U effect against P-gp activity in MDR NSCLC cells. As a model system, we used sensitive NCI-H460 cells without P-gp expression and their MDR counterpart–NCI-H460/R cells with P-gp expression. Our investigation was directed towards revealing cellular processes that explain the delayed decrease in P-gp activity after Coleon U treatment because we showed that Coleon U does not directly interact with P-gp [17].

## 2. Materials and Methods

### 2.1. Reagents

RPMI 1640 medium and DMEM (Dulbecco’s Modified Eagle Medium) were acquired from Capricorn Scientific GmbH (Ebsdorfergrund, Germany). L-glutamine, trypsin, and penicillin–streptomycin solution were from Biowest (Nuaillé, France). Thiazolyl blue tetrazolium bromide (MTT), fetal bovine serum (FBS), DMSO, Hoechst 33342, and carboxyfluorescein succinimidyl ester (CFSE) were purchased from Thermo Fisher Scientific (Waltham, MA, USA). An Apoptosis Detection Kit based on Annexin-V-FITC (AV) and propidium iodide (PI) staining was acquired from Abcam (Cambridge, UK). Bovine serum albumin (BSA) was obtained from Serva (Heidelberg, Germany). Rhodamine 123 (Rho123) and tetramethylrhodamine ethyl ester (TMRE) were purchased from Sigma (St Louis, MO, USA). Carbonyl cyanide m-chlorophenyl hydrazine (CCCP, C2759) was purchased from Sigma-Aldrich (Darmstadt, Germany). FITC-conjugated anti-P-gp antibody was obtained from BD Biosciences (Winnersh, Berkshire, UK). Anti-P-gp mouse monoclonal antibody (Abcam, Cambridge, UK; ab10333) and secondary antibodies Alexa Fluor 555 goat anti-mouse secondary antibody (#A-21422, Thermo Fisher Scientific, Waltham, MA, USA) were obtained.

### 2.2. Drugs

Coleon U compound, isolated from *Plectranthus mutabilis* Codd. [17], was dissolved in dimethyl sulfoxide (DMSO) as a 20 mM aliquot and kept at room temperature until use. Verapamil (Sigma-Aldrich Chemie Gmbh, Hamburg, Germany) and Sodium Vanadate (Na_3_VO_4_) (Promega, Madison, WI, USA) were stored as 10 mM aliquots at −20 °C. Paclitaxel—PTX (Sigma-Aldrich Chemie Gmbh, Hamburg, Germany) was diluted in absolute ethanol and 1 mM aliquots were stored at −20 °C. Before treatment, all drugs were freshly diluted in sterile water.

### 2.3. Cell Culture

NSCLC NCI-H460 cell line was purchased from the American Type Culture Collection (Rockville, MD, USA). P-gp overexpressing MDR NCI-H460/R cells were selected by continuous exposure to stepwise increasing concentrations of DOX from NCI-H460 cells [21]. NCI-H460/R and their sensitive counterparts were maintained in RPMI 1640 medium supplemented with 10% FBS, 2 mM L-glutamine, and 10,000 U/mL penicillin, 10 mg/mL streptomycin, 25 mg/mL amphotericin B solutions. Normal human embryonic pulmonary fibroblasts-MRC-5 cells were cultured in DMEM supplemented with 10% FBS, 4 g/L glucose, 2 mM L-glutamine, 5000 U/mL penicillin, and 5 mg/mL streptomycin solution. All cell lines were subcultured at 72 h intervals using 0.25% trypsin/EDTA and seeded into a fresh medium at 16,000 cells/cm^2^. All cell lines were maintained at 37 °C in a humidified 5% CO_2_ atmosphere.

### 2.4. MTT Assay 

Cell viability was assessed by the MTT assay (AppliChem GmbH, Darmstadt, Germany). NCI-H460, NCI-H460/R, and MRC-5 cells grown in 25 cm^2^ tissue flasks were trypsinized, and 2000 cells/well were seeded into flat-bottomed 96-well tissue culture plates following the overnight incubation. Subsequently, the cells were treated with increasing concentrations of Coleon U (2, 5, 10, 20, and 50 µM) for 72 h. At the end of the incubation period, MTT was added to each well in a final concentration of 0.2 mg/mL for 4 h. MTT-containing medium was then removed and formazan product was dissolved in 200 µL of dimethyl sulfoxide, and the absorbance was measured at 570 nm using an automatic Multiskan Sky reader (Thermo Scientific, Waltham, MA, USA).

### 2.5. CFSE Proliferation Assay

CFSE proliferation assay is based on the ability of the carboxyfluorescein succinimidyl ester (CFSE) dye to covalently bind free amines of the intracellular molecules via its succinimidyl group. CFSE is cell permeable and has no significant effect on their proliferative capacity. It enables the monitoring of cells over a period of 15 divisions [22]. The fluorescence intensity of CFSE, which gradually declines during cell divisions, enables the assessment of cell proliferation rates in treated versus untreated cells. For CFSE labeling, NCI-H460 and NCI-H460/R were incubated for 15 min in 0.1% FBS/PBS solution containing 1 μM CFSE at 37 °C, after which the cells were washed three times with PBS. Next, the CFSE-treated cells were seeded in 6-well plates, accommodated overnight, and then treated with Coleon U (7.5 µM, 15 µM, and 30 µM). PTX (100 nM in NCI-H460 and 2 µM in NCI-H460/R) was used as a positive control. After 48 h and 72 h, the cells were trypsinized and washed in ice-cold PBS and finally resuspended in 500 µL PBS. The fluorescence intensities of at least 20,000 cells per sample were detected on CytoFLEX flow cytometer (Beckman Coulter, Indianapolis, IN, USA) using fluorescence channel 1 (FL1) at 525 nm. The results were analyzed in Summit v4.3 software (Dako Colorado Inc., Fort Collins, CO, USA) using subtraction analysis.

### 2.6. Cell Death Analysis by Flow Cytometry

Induction of cell death after Coleon U treatment was assessed by Abcam Apoptosis Detection Kit through dual staining with Annexin V—FITC/Propidium Iodide (AV/PI) according to the manufacturer’s instructions. NCI-H460 and NCI-H460/R cells were seeded in adherent 6-well plates at a density of 50,000 cells/well and incubated overnight. Both cell lines were treated with Coleon U (15, and 30 µM). Treatment with 500 nM PTX was used as a positive control in both cell lines. After 72 h, both attached and floating cells were collected and washed in PBS. After centrifugation, cells were resuspended in 100 µL of binding buffer containing AV and PI (1:1 ratio), and incubated for 15 min at room temperature in the dark. Cells were then analyzed on a CytoFLEX flow cytometer (Beckman Coulter, Indianapolis, IN, USA). The fluorescence intensities of AV and PI were measured in green fluorescence channel 1 (FL1) at 525 nm and red fluorescence channel 2 (FL2) at 585 nm, respectively. In each sample, the acquisition of 20,000 cells was performed, and the percentages of viable (AV− PI−), early apoptotic (AV+ PI−), late apoptotic (AV+ PI+), and necrotic (AV− PI+) cells were analyzed by CytExpert 2.4 software (Beckman Coulter, Indianapolis, IN, USA).

### 2.7. Rho123 Accumulation Assay

The accumulation of Rho123 was analyzed by flow cytometry utilizing the ability of Rho123 as a P-gp substrate to emit fluorescence. The intensity of the fluorescence is proportional to Rho123 accumulation [23]. NCI-H460/R cells were seeded in 6-well plates and grown overnight. Cells were treated with Coleon U (5 μM and 10 μM), verapamil (5 μM), or Na_3_VO_4_ (1 µM), in single treatments or in combined treatments of Coleon U (5 μM and 10 μM) or Verapamil (5 μM) with Na_3_VO_4_ (1 μM). The cells were incubated for 30 min and 72 h. At the end of incubation periods, Rho123 (2.5 µM) was added and the cells were further incubated for 30 min at 5% CO_2_ atmosphere at 37 °C. After the accumulation period, the cells were pelleted by centrifugation, washed with cold PBS, and kept on ice in the dark until the analysis. The samples were analyzed on a CytoFLEX flow cytometer (Beckman Coulter, Indianapolis, IN, USA). The orange fluorescence of Rho123 was assessed on fluorescence channel 1 (FL1) at 525 nm. A minimum of 10,000 events were assayed for each sample. The obtained mean fluorescence intensities were analyzed in Summit v4.3 software (Dako Colorado Inc., Fort Collins, CO, USA).

### 2.8. Mitochondrial Membrane Potential Analysis

To analyze the effect of Coleon U on mitochondrial membrane potential, an early marker for apoptosis, TMRE dye, was used [24]. TMRE is a cell membrane permeable, fluorescent dye that accumulates in intact mitochondria. Depolarized or inactive mitochondria exhibit decreased membrane potential, resulting in reduced TMRE accumulation. NCI-H460/R cells were seeded in 6-well plates at a density of 50,000 cells/well. After 24 h, cells were treated with Coleon U (5 µM and 10 µM), verapamil (5 µM), and Na_3_VO_4_ (1 µM). Cells were incubated for an additional period of 72 h. CCCP was used as a positive control due to its notable depolarizing effect caused by the uncoupling of the proton gradient in the inner mitochondrial membrane [25]. The cells were treated with 10 µM CCCP 30 min prior to TMRE staining. For TMRE staining the cells were trypsinized, resuspended in RPMI media containing 500 nM TMRE, and incubated for 30 min at 37 °C in the dark. After washing twice in PBS, the red fluorescence emission of TMRE was immediately detected in the FL2 channel on a CytoFLEX flow cytometer (Beckman Coulter, Indianapolis, IN, USA). A minimum of 20,000 events were assayed per sample. The obtained data were analyzed in Summit v4.3 software (Dako Colorado Inc., Fort Collins, CO, USA), using subtraction analysis.

### 2.9. Flow Cytometric Analysis of P-gp Expression

P-gp expression level in NCI-H460/R cells was assessed by flow cytometry. Cells were seeded in adherent 6-well plates and treated with Coleon U (5 and 10 µM) for 72 h. At the end of the incubation period, cells were collected by trypsinization, washed with PBS, and directly immunostained with FITC-conjugated anti-P-gp antibody according to the manufacturer’s protocol. The samples were kept in the dark until the analysis on a CytoFLEX flow cytometer (Beckman Coulter, Indianapolis, IN, USA). The fluorescence of FITC-conjugated anti-P-gp was detected on fluorescence channel 1 (FL1) at 525 nm. A minimum of 10,000 events were collected for each sample (the gate excluded cell debris and dead cells). The obtained results were analyzed by Summit v4.3 software (Dako Colorado Inc., Fort Collins, CO, USA) using subtraction analysis.

### 2.10. Immunocytochemistry 

Immunocytochemistry was used for the quantification of the P-gp expression. For immunostaining, NCI-H460/R cells were seeded into 8-well chamber slides (Nunc, Nalgene, Roskilde, Denmark) at a density of 25,000 cells/chamber. Cells were allowed to attach to the surface overnight before the treatment with 30 µM Coleon U. In addition, NCI-H460/R cells were treated with PTX (500 nM), which served as a positive control for the induction of P-gp expression. After 72 h, cells were fixed with 4% paraformaldehyde for 20 min at RT and washed three times with PBS. Cells were then blocked with 2% bovine serum albumin (BSA) in PBS for 1 h at room temperature (RT). The anti-P-gp mouse monoclonal antibody was diluted 1:1000 in 2% BSA in PBS and incubated with the cells at 4 °C overnight. The cells were washed three times with PBS before adding Alexa Fluor 555 goat anti-mouse secondary antibody diluted 1:1000 in 2% BSA in PBS. The secondary antibody was incubated with the cells at RT for 2 h in the dark. To label the nuclei, the cells were incubated for 2 h in the dark with Hoechst 33342 at a final concentration of 1 µg/mL at RT. Finally, cells were washed three times with PBS, mounted in Mowiol, and stored at 4 °C in the dark before imaging. Fluorescently labeled cells were imaged using the ImageXpress^®^ Pico Automated Cell Imaging System (Molecular Devices, San Jose, CA, USA). Analysis of the obtained images was performed using CellReporterXpress 2.9 software (Molecular Devices) with the Cell Scoring Analysis Protocol.

### 2.11. SwissADME Online Tool

WLOGP (water partition coefficient, a measure of the lipophilicity of a molecule) and TPSA (topological polar surface area, a measure of the surface area of a molecule that is polar) values for Coleon U and verapamil were generated using the SwissADME website [26,27]. The generated “boiled-egg” illustrates the position of the molecules in the WLOGP-versus-TPSA plot and enables the evaluation of passive gastrointestinal absorption and brain penetration of small molecules such as Coleon U and verapamil. The white region indicates a high probability of passive absorption by the human intestine (HIA), and the yellow region (yolk) indicates a high probability of blood–brain barrier (BBB) penetration. Yolk and white areas are not mutually exclusive. Points colored in blue show molecules predicted to be actively extruded by P-gp (PGP+) and are in red if predicted as nonsubstrate of P-gp (PGP−).

### 2.12. Statistical Analyses

For the results obtained by MTT assay, a nonparametric Kruskal–Wallis multiple comparisons test was applied, while IC_50_ values were calculated by nonlinear regression analysis using GraphPad Prism 8.0.2 for Windows (San Diego, CA, USA). The results obtained by CFSE, Rho123 accumulation, mitochondrial membrane potential, and immunocytochemistry were analyzed by GraphPad Prism 8.0.2 (San Diego, CA, USA) using two-way-ANOVA Sidak’s multiple comparisons test. For the cell death analysis, the results were analyzed by GraphPad Prism 8.0.2 (San Diego, CA, USA) using two-way-ANOVA Dunnett’s multiple comparisons test.

## 3. Results and Discussion

### 3.1. Sensitivity of NSCLC Cells to Coleon U (Effects on Cell Viability, Proliferation, and Cell Death Induction)

**Figure 1:**
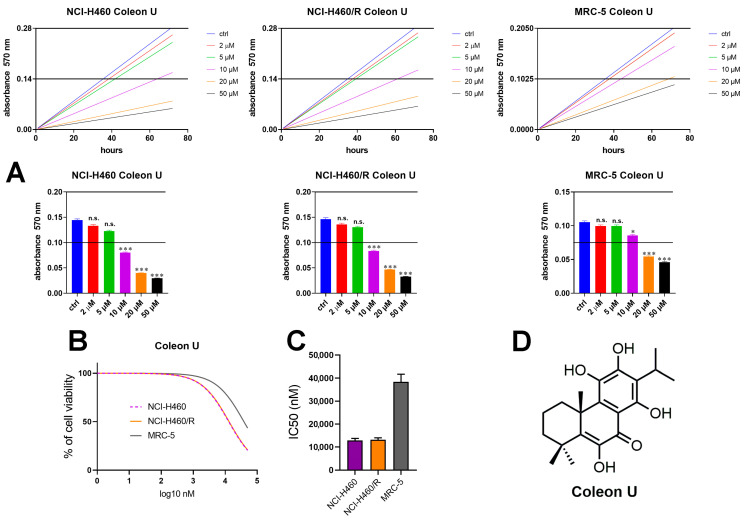
Concentration-dependent effect on cell viability upon Coleon U treatment. (**A**) The effects of different Coleon U concentrations (2, 5, 10, 20, and 50 µM) on the absorbance increase detected in NCI-H460, NCI-H460/R, and MRC-5 between time points 0 h and 72 h assessed by MTT presented as linear graphs and corresponding bar graphs with statistical analysis. The experiments were performed three times (n = 3). The statistically significant difference compared to the corresponding untreated controls: n.s. (nonsignificant), * (*p* ˂ 0.05), *** (*p* ˂ 0.001). (**B**) The cell viability was illustrated by nonlinear regression analysis; (**C**) the IC_50_ values obtained in NCI-H460, NCI-H460/R, and MRC-5. Mean values ± SEM were calculated from three independent experiments (n = 3). (**D**) Coleon U chemical structure.

The effects of Coleon U on cell viability were evaluated by MTT assay, which detects viable mitochondria (Figure 1). Therefore, the cell viability sensing of the MTT assay is due to preserved mitochondrial membrane electron transport chain [28]. The results obtained after 0 h and 72 h treatments in NSCLC NCI-H460, NCI-H460/R cell lines, and normal human embryonic pulmonary fibroblasts (MRC-5) show that the greatest reduction in cancer cell viability in the NCI-H460 and NCI-H460/R cell lines was observed with three concentrations of Coleon U (10, 20, and 50 μM), while the greatest reduction in cell viability in normal MRC-5 cells was achieved with the two highest concentrations of Coleon U (20 and 50 μM), as shown in Figure 1A. These concentrations that were at or below the threshold (half maximum absorbance, Figure 1A) were considered cytotoxic for each tested cell line.

Importantly, Coleon U exerted a similar inhibitory effect on cell viability in sensitive and MDR NSCLC cell lines with similar IC_50_ values (Figure 1B,C). This indicates that the MDR phenotype of NCI-H460/R cells did not reduce the effectiveness of Coleon U, which should be considered as a favorable feature of the potential anticancer compound. Additionally, the IC_50_ value determined for the normal MRC-5 cells was threefold higher compared with the IC_50_ values obtained in the cancer cell lines. Therefore, Coleon U has a greater inhibitory effect on the cancer cells compared to the normal cells, implying its selectivity towards cancer cells. 

**Figure 2:**
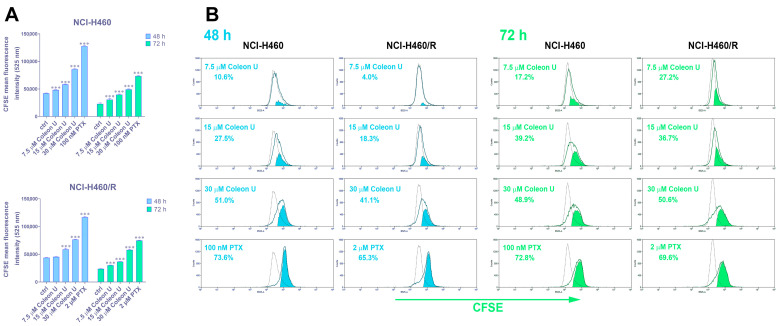
The antiproliferative effect of Coleon U. (**A**) The histograms show the increases in CFSE fluorescence intensity as a measure of sustained cell proliferation. The effect of different Coleon U concentrations (7.5, 15, and 30 µM) was evaluated after 48 h and 72 h in NCI-H460 and NCI-H460/R cells. PTX (100 nM in NCI-H460 and 2 µM in NCI-H460/R) was used as a positive control. The dotted line represents untreated control. Values are presented as mean ± SEM (n = 3). The statistically significant difference between the treated and control groups is shown as *** (*p* < 0.001). (**B**) Representative flow cytometric profiles of CFSE-labeled NCI-H460 and NCI-H460/R cells treated with Coleon U and PTX, evaluated after 48 h and 72 h, are shown.

Other authors identified Coleon U as a selective activator of protein kinase C-δ [20], whose activity is related to antiproliferative effects [29]. Therefore, we assessed the antiproliferative effects of Coleon U after 48 h and 72 h by CSFE in NCI-H460 and NCI-H460/R cells (the live cell population was gated during acquisition on the flow cytometer). Cells were treated with three concentrations of Coleon U identified according to MTT assay: a noncytotoxic concentration of 7.5 µM, and two cytotoxic concentrations of 15 µM (approximately IC_50_) and 30 µM (2 × IC_50_). The results showed that treatment with 7.5 µM Coleon U led to a significant increase in CFSE fluorescence in both cell lines after 72 h (Figure 2A,B). Other treatments (15 µM and 30 µM) significantly sustained the proliferation of the non-small-cell lung carcinoma cells after 48 h and 72 h (Figure 2A,B). Obtained results also showed that there was a significant decrease in cell proliferation in both cell lines after treatment with PTX (100 nM in NCI-H460 and 2 µM in NCI-H460/R). Thus, Coleon U at 7.5 µM and 15 µM showed a time-dependent antiproliferative effect, while the effect of 30 µM was similar after 48 h and 72 h. Likewise, the PTX effect was not time-dependent (Figure 2A,B).

**Figure 3:**
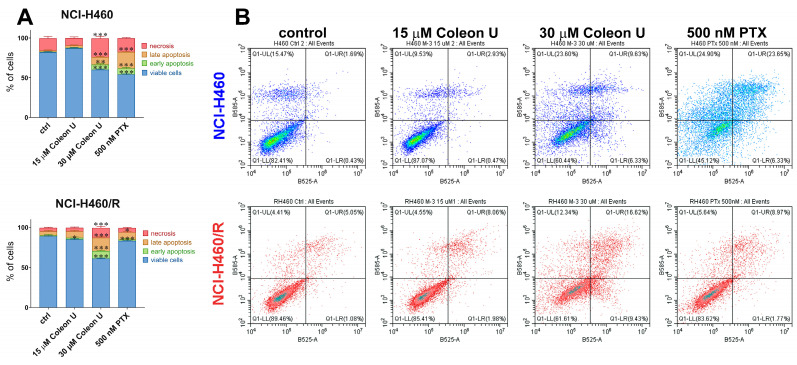
Cell death induction by Coleon U. AV/PI staining was used to assess cell death in NCI-H460 and NCI-H460/R cells following 72 h treatments with Coleon U (15 and 30 µM). PTX (500 nM) was used as a positive control. (**A**) Histograms show the percentage of viable (AV− PI−), early apoptotic (AV+ PI−), late apoptotic (AV+ PI+), and necrotic (AV− PI+) cells after treatments. The experiments were performed three times (n = 3). The statistically significant difference compared to the corresponding untreated controls: * (*p* ˂ 0.05), ** (*p* ˂ 0.01), *** (*p* ˂ 0.001). (**B**) Representative flow cytometric profiles of AV/PI-labeled cells are shown for each treatment. Coleon U is nominated as M-3.

To examine whether the induction of cell death contributes to the NSCLC cells’ sensitivity to Coleon U, 15 µM and 30 µM treatments in NCI-H460 and NCI-H460/R were assessed by Annexin-V-FITC/Propidium Iodide staining after 72 h (Figure 3). The results showed that treatment with 30 µM Coleon U significantly increased the proportion of necrotic, late apoptotic, and early apoptotic NCI-H460 and NCI-H460/R cells compared to controls (Figure 3A,B). We reconfirmed that the potential of Coleon U to induce cell death was not compromised by the MDR phenotype of NCI-H460/R. However, the effect of 15 µM Coleon U was less pronounced, indicating that this concentration is not cytotoxic, as was previously suggested by the MTT assay. Therefore, the MTT can be considered the most sensitive method for the evaluation of Coleon U effects. It should be noted that the percentage of dying cells in the MDR NCI-H460/R cell line was significantly higher after 30 µM Coleon U treatment, compared with 500 nM PTX, and that the resistance to PTX was clearly mirrored in different efficacy in NCI-H460 and NCI-H460/R cells (Figure 3A,B).

### 3.2. Coleon U Mechanisms Involved in P-gp Activity Modulation

**Figure 4:**
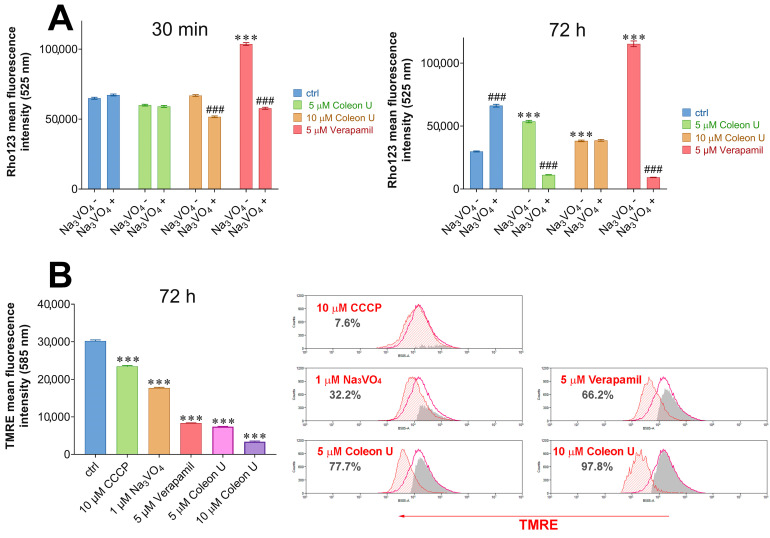
The effects of Coleon U on P-gp function and mitochondrial membrane potential in NCI-H460/R cells. (**A**) P-gp function in MDR NCI-H460/R cells was evaluated after 30 min and 72 h in individual treatments with Coleon U (5 and 10 µM), verapamil (5 µM), and sodium orthovanadate-Na_3_VO_4_ (1 µM) or in combined treatments of Coleon U or verapamil with sodium orthovanadate-Na_3_VO_4_. The histograms show Rho123 accumulation in treated NCI-H460/R cells. Values are presented as mean ± SEM (n = 3). The statistically significant difference between the treated and control groups is shown as *** (*p* < 0.001). The statistically significant difference between single treatment and corresponding combined treatment is shown as ### (*p* < 0.001). (**B**) Coleon U triggers loss of mitochondrial membrane potential in MDR NCI-H460/R cells. Mitochondrial membrane potential in NCI-H460/R cells was evaluated after 72 h treatments with Coleon U (5 and 10 µM), verapamil (5 µM), and sodium orthovanadate-Na_3_VO_4_ (1 µM) using TMRE staining. CCCP was used as a positive control. Histograms in the left panel represent the average fluorescence intensity of TMRE from three independent experiments. The statistically significant difference between the treated and control groups is shown as *** (*p* < 0.001). Representative flow cytometric profiles for each condition are shown in the right panel.

P-gp protein, found in MDR cancer cells, reduces the concentration of certain drugs inside the cells due to its transporter activity mediated by ATP hydrolysis. Therefore, the P-gp overexpression and activity significantly enhance the cellular ATP requirements [30]. In our previous study, Coleon U exerted a delayed effect on P-gp activity by decreasing it after 72 h and reversing doxorubicin resistance in the subsequent treatment [17]. To investigate how Coleon U affects P-gp activity, we conducted experiments using well-known inhibitors of this pump, sodium orthovanadate (Na_3_VO_4_), and verapamil. Sodium orthovanadate directly inhibits plasma membrane ATPases, including P-gp, and has shown anticancer activity against several types of cancer [31,32]. On the other hand, verapamil, a first-generation P-gp inhibitor, is a P-gp substrate that stimulates the ATPase activity of P-gp [33]. Verapamil promotes intracellular accumulation of the drug when applied with chemotherapeutic agents, as shown in various cancer cell lines: NSCLC, colorectal cancer, leukemia, and neuroblastoma [34,35,36]. To evaluate the ability of Coleon U to inhibit the P-gp transporter, a Rho123 accumulation assay was performed. Resistant NSCLC cells (NCI-H460/R) expressing P-gp, were treated with Coleon U (5 μM and 10 μM) and with verapamil (5 μM), in single treatments or in combination with sodium orthovanadate (1 μM). The treatments were conducted at two different time points: 30 min for the evaluation of the direct interaction with P-gp and 72 h for the evaluation of the indirect effect on P-gp activity such as effects on the P-gp expression [37], related proteins involved in the P-gp expression regulation [38], changes in the intracellular pH [39], or availability of ATP as a fuel molecule for the P-gp functioning [36]. Verapamil at 5 μM significantly inhibited the efflux of Rho123 in NCI-H460/R cells after 30 min, while Coleon U at both concentrations of 5 μM and 10 μM and sodium orthovanadate at 1 μM were not effective, as shown in Figure 4A. Sodium orthovanadate significantly decreased the level of Rho123 in combination with verapamil and Coleon U (10 μM) (Figure 4A). After 72 h, sodium orthovanadate (1 μM), verapamil (5 μM), and Coleon U (5 μM and 10 μM) increased the accumulation of Rho123 in NCI-H460/R cells. This suggests that verapamil directly interacts with P-gp as a P-gp substrate and sustains this effect during 72 h as an ATPase stimulator, while sodium orthovanadate and Coleon U effects on Rho123 intracellular accumulation are evident later, after 72 h. When NCI-H460/R cells were treated with combinations of verapamil or Coleon U with sodium orthovanadate, a decrease in the fluorescent Rho123 signal was observed after 72 h (Figure 4A). This suggests an antagonistic effect between verapamil (an ATPase stimulator) and sodium orthovanadate (an ATPase inhibitor) as well as Coleon U and sodium orthovanadate. Therefore, we assumed that Coleon U interferes with the ATP metabolism, probably its production in mitochondria. This is also supported by the fact that the MTT assay, which detects viable mitochondria, was more powerful in sensing Coleon U effects than AV/PI assay, which discriminates viable from dead cells.

The inner mitochondrial membrane is essential for the generation of ATP via the mitochondrial respiratory chain. When the mitochondrial membrane potential is depolarized, the respiratory chain is blocked, leading to a reduced efficiency of ATP production [30]. Decreased intracellular ATP levels also can affect the functioning of the P-gp, as an ATP-dependent membrane transporter [30]. Thus, the compound Coleon U was examined for its effect on the disruption of mitochondrial potential in MDR NCI-H460/R cells. The TMRE labeling was used to assess changes in mitochondrial membrane potential. The results showed that 72 h after treatment with Coleon U (5 µM and 10 µM), TMRE fluorescence intensities were significantly decreased compared with the control (Figure 4B). This suggests that Coleon U induced notable depolarization of the mitochondrial membrane in NCI-H460/R. Treatments with sodium orthovanadate (1 μM) and verapamil (5 μM) also significantly lowered the TMRE signal in NCI-H460/R cells. The obtained results are consistent with previous findings in the literature that sodium orthovanadate decreases mitochondrial membrane potential and ATPase activity in sorafenib-resistant hepatocellular carcinoma cells, ultimately promoting apoptosis [32]. Our results suggest that Coleon U induces depolarization of the mitochondrial membrane potential in MDR NCI-H460/R cancer cells that can cause a decrease in ATP production, indirectly affecting P-gp function. Furthermore, the disruption of mitochondrial function caused by Coleon U can potentially contribute to the activation of cell death pathways.

**Figure 5:**
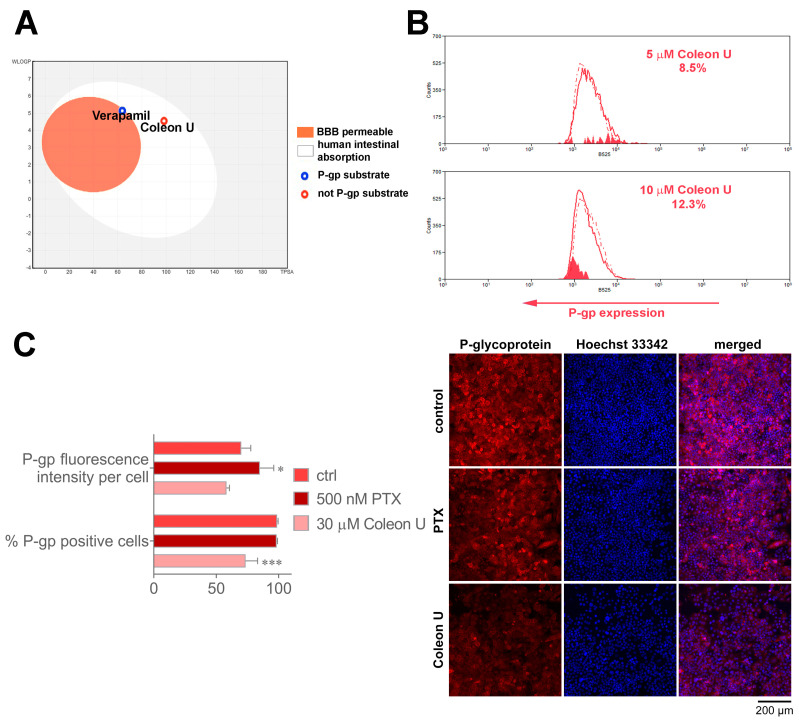
The effects of Coleon U on P-gp expression. (**A**) The “boiled-egg” plot – Brain or IntestinaL EstimateD permeation predictive model (plot of WLOGP against TPSA) of Coleon U and verapamil from SwissADME online tool. The points in yellow (boiled-egg’s yolk) represent molecules predicted to passively permeate through the blood–brain barrier (BBB), while the points in white (boiled-egg’s white) represent molecules predicted to be passively absorbed by the gastrointestinal tract (HIA). Blue dots represent molecules predicted to be P-gp substrates, while red dots are molecules predicted not to be substrates for P-gp. (**B**) Flow cytometric profiles of P-gp expression in NCI-H460/R cells treated with Coleon U for 72 h treatment. (**C**) The histograms in the left panel show P-gp fluorescence intensity per cell and percent of P-gp positive NCI-H460/R cells treated with Coleon U and PTX for 72 h. Values are presented as mean ± SEM (n = 3). The statistically significant difference between the treated and control groups is shown as * (*p* ˂ 0.05) and *** (*p* < 0.001). Representative immunofluorescence micrographs of anti-P-gp labeled NCI-H460/R cells are shown in the right panel. Nuclei were counterstained with Hoechst 33342. Scale bar = 200 μm.

Although the results obtained by MTT, CFSE, and AV/PI showed no significant difference in Coleon U efficacy between NCI-H460 and NCI-H460/R cells, which means that Coleon U efficacy is not affected by MDR and P-gp activity, we performed SwissADME analysis to ensure that Coleon U is not a P-gp substrate. SwissADME results illustrated as “boiled egg” [27] showed that Coleon U can be easily absorbed by the human intestine and confirmed that Coleon U is not a P-gp substrate, while verapamil is a P-gp substrate (Figure 5A). To examine the above mechanisms that may lead to a decrease in P-gp expression and contribute to the observed delayed effect of Coleon U on P-gp activity, we studied P-gp expression in NCI-H460/R cells after 72 h treatment with Coleon U (5 and 10 μM), as shown in Figure 5B,C. Analysis of P-gp expression by flow cytometry revealed that Coleon U tended to decrease P-gp expression in NCI-H460/R cells. Specifically, treatment with 5 μM resulted in an approximate 9% decrease in P-gp expression, while treatment with 10 μM led to an approximately 12% decrease in P-gp expression (Figure 5B). We also examined whether the cytotoxic concentration of Coleon U (30 μM) affects P-gp expression in NCI-H460/R cells (Figure 5C). To that end, we used fluorescent labeling of P-gp and analysis performed on the ImageXpress^®^ Pico Automated Cell Imaging System. The results obtained by CellReporterXpress software revealed that 30 μM Coleon U significantly reduced the portion of NCI-H460/R cells expressing P-gp without changing the level of expression per cell (Figure 5C). This suggests that Coleon U exerts its cytotoxic effect on P-gp-positive cells and does not induce P-gp expression. Therefore, Coleon U has valuable characteristics for the treatment of MDR cancers. In contrast, treatment of NCI-H460/R cells with PTX resulted in a significant increase in P-gp fluorescence intensity per cell, while the portion of P-gp cells was not changed (Figure 5C). This suggests a continuation of the P-gp upregulation upon PTX treatment, while Coleon U may hold promise in overcoming MDR by modulating both P-gp activity and expression.

## 4. Conclusions

Coleon U is a natural compound with a significant anticancer potential whose activity against MDR cancers has not been truly investigated. Although the activation of protein kinase C-δ (that promotes antiproliferative and proapoptotic effects) was identified as the Coleon U mechanism of anticancer action [20], the literature data on Coleon U as an anticancer agent is rare. It seems that the anticancer potential of Coleon U has not attracted the attention of the scientific community enough. Herein, we showed that Coleon U is not a P-gp substrate and does not directly interact with P-gp (an ATP-dependent transporter). However, it decreases P-gp activity after 72 h and this delayed effect is accompanied by a significant mitochondrial membrane potential decrease, suggesting the exhaustion of the ATP necessary for the P-gp activity. Moreover, we showed that sodium orthovanadate, which is an ATPase inhibitor, antagonizes the effect of verapamil, an ATPase stimulator, while the same antagonizing effect of sodium orthovanadate is achieved in combination with Coleon U. An important finding is the tendency of Coleon U to decrease the P-gp expression, which also qualifies Coleon U as a valuable modulator of MDR mediated by P-gp expression and activity. In our recent study, we showed the sensitizing effect of Coleon U in the subsequent combination with doxorubicin in MDR cancer cells [17]. Our work may stimulate other studies on different natural compounds that interfere with ATP production for the identification of novel P-gp modulators.

## Data Availability

The data presented in this study are available on request from the corresponding author. The data are not publicly available because our institutional repository is not yet ready to collect experimental data.
